# Pyocyanin-induced mucin production is associated with redox modification of FOXA2

**DOI:** 10.1186/1465-9921-14-82

**Published:** 2013-08-05

**Authors:** Yonghua Hao, Zhizhou Kuang, Ying Xu, Brent E Walling, Gee W Lau

**Affiliations:** 1 Department of Pathobiology, University of Illinois at Urbana-Champaign 2001, Lincoln Avenue, Urbana, IL, 61802, United States of America; 2 Laboratory of Clinical Immunology, Sun Yat-Sen Memorial Hospital, Sun Yat-Sen University, Guangzhou, Guangdong, People’s Republic of China

**Keywords:** Pyocyanin, Reactive oxygen and nitrogen species, Posttranslational modifications, FOXA2, Mucus hypersecretion

## Abstract

**Background:**

The redox-active pyocyanin (PCN) is a toxic, secondary metabolite secreted by the respiratory pathogen *Pseudomonas aeruginosa (PA)*. Previously, we have shown that mouse lungs chronically exposed to PCN develop goblet cell hyperplasia and metaplasia (GCHM) and mucus hypersecretion, fibrosis and emphysema. These pathological features are commonly found in the airways of several chronic lung diseases, including cystic fibrosis (CF), as well as in mouse airways deficient in the forkhead box A2 (FOXA2), a transcriptional repressor of goblet GCHM and mucus biosynthesis. Furthermore, PCN inhibits FOXA2 by activating the pro-GCHM signaling pathways Stat6 and EGFR. However, it is not known whether PCN-generated reactive oxygen (ROS) and nitrogen (RNS) species posttranslationally modify and inactivate FOXA2.

**Methods:**

We examined the posttranslational modifications of FOXA2 by PCN using specific antibodies against oxidation, nitrosylation, acetylation and ubiquitination. Electrophoretic mobility shift assay (EMSA) was used to examine the ability of modified FOXA2 to bind the promoter of *MUC5B* mucin gene. In addition, we used quantitative real time PCR, ELISA, immunofluorescence and mouse lung infection to assess whether the loss of FOXA2 function caused GCHM and mucin overexpression. Finally, we examined the restoration of FOXA2 function by the antioxidant glutathione (GSH).

**Results:**

We found that PCN-generated ROS/RNS caused nitrosylation, acetylation, ubiquitination and degradation of FOXA2. Modified FOXA2 had reduced ability to bind the promoter of the *MUC5B* gene. The antioxidant GSH alleviated the modification of FOXA2 by PCN, and inhibited the overexpression of MUC5AC and MUC5B mucins.

**Conclusion:**

These results suggest that PCN-mediated posttranslational modifications of FOXA2 are positively correlated with GCHM and overexpression of airway mucins. Furthermore, antioxidant treatment restores the function of FOXA2 to attenuate GCHM and mucus hypersecretion.

## Background

*Pseudomonas aeruginosa (PA)* is an important pathogen of patients with cystic fibrosis (CF) and non-CF bronchiectasis, and chronic obstructive pulmonary disease (COPD) [1-3]. *PA* infection is associated with more sputum, extensive bronchiectasis, increased hospitalizations and worse quality of life. *PA* elaborates multiple virulence factors to thrive in the mucus-rich airways [4]. However, at chronic stage, *PA* alters its virulence [4,5], by repressing the expression of flagella [6], mutating the immunogenic O-antigen of LPS [7], overproducing the mucoid alginate [8] and switching to the biofilm mode of growth [9]. However, alginate is poorly immunogenic [10]. *PA* factors that are still secreted abundantly include the quorum-sensing effectors homoserine lactones and quinolones, which regulate biofilm formation [4,5]. However, at approximately 20 nM concentration found within CF airways, these effectors are thought to be non-toxic [4]. Another important *PA* factor is the redox-active exotoxin pyocyanin (PCN) [4,11]. A previous study involving limited sputum samples from CF and non-CF bronchiectatic patients had recovered 16.5 and 27 μg/ml of PCN, respectively [12]. Importantly, *PA* increases PCN production when cultured in medium supplemented with CF sputum [13].

PCN redox cycles and forms ROS [11]. PCN-generated O_2_^-^ can react with NO to form RNS, including the highly toxic peroxynitrite. ROS/RNS damage host targets (DNA, lipid, proteins) and modulate cellular and inflammatory functions [14]. PCN depletes GSH in cultured airway epithelial cells [15] and inactivates catalase [16]. Excessive ROS/RNS production and inhibition of antioxidative mechanisms by PCN overwhelm the antioxidant capacity of the tissue, leading to lung damage. PCN damages ciliated epithelium and inhibits mucus transport [12], induces bronchoconstriction [17], and decreases trachea mucus velocity [18,19]. Furthermore, PCN inhibits NO production in macrophages and endothelial cells [20], prostacyclin production by endothelial cells [21], oxidation of leukotriene B4 by neutrophils [22], eicosanoid metabolism by platelets [23], and production of IL-2 and the IL-2 receptor in T cells [24]. PCN has opposite effects on airway epithelial cells, inhibiting the release of RANTES and MCP-1 while stimulating Ca^2+^ signaling and IL-8 release [25,26]. Finally, PCN inactivates α1-protease inhibitor [27] and causes apoptosis in neutrophils [28]. Antioxidants detoxify PCN, suggesting that its virulence is redox dependent [22,26,28,29].

Importantly, we have shown that PCN is important for both acute and chronic lung infections [30,31]. GCHM, excessive mucus secretion and defective mucociliary clearance, airway obstruction, bacterial infection, and neutrophilic infiltration are important clinical features of CF and other chronic airway diseases [1-3]. We have shown that mouse lungs chronically exposed to PCN undergo remodeling characterized by over-proliferation of goblet cells in large bronchi and terminal bronchioles, emphysema, fibrosis, and an influx of immune cells [31]. These pathological features resemble the airways of FOXA2^−/−^ mice [32], as well as the CF and COPD airways chronically infected by *PA*. Importantly, we have shown that PCN inhibits FOXA2 expression by activating the pro-GCHM signaling pathways Stat6 and EGFR [31,33]. In this study, we tested the hypothesis that PCN-generated ROS/RNS posttranslationally modify FOXA2, disabling its ability to regulate GCHM and mucin expression.

## Materials and methods

### PCN and chemicals

All chemicals, including PCN were purchased from Sigma Chemical Co., unless stated otherwise. Chemically synthesized PCN (Sigma, #R9532) (St. Louis, MO, USA) is preferred over PCN purified from *PA* cultures to eliminate any contaminants (e.g., LPS, CpG DNA), which may cause lung injuries. PCN was resuspended to 1 μg/ml in sterile H_2_O.

### Cell cultures

**Figure 1:**
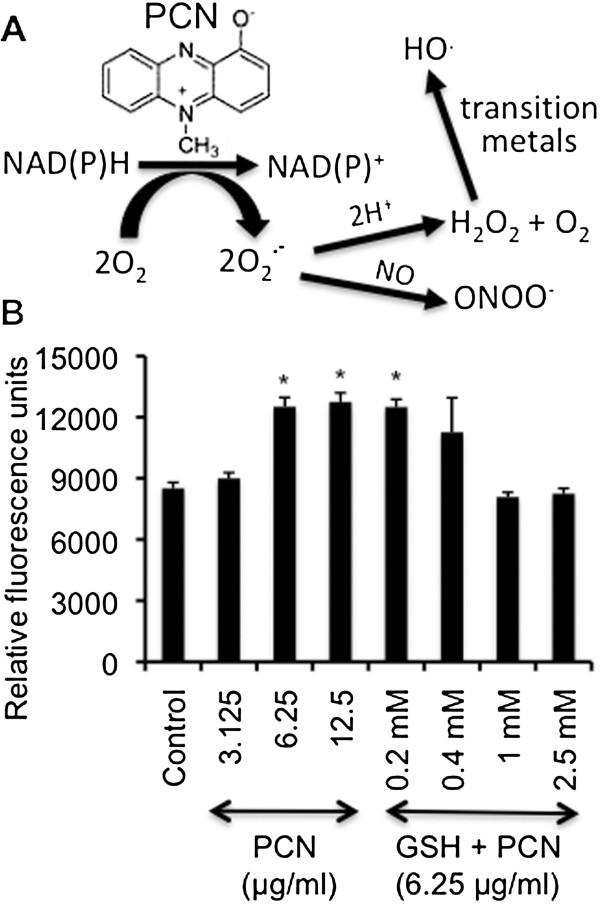
**PCN causes oxidative stress in NCI-H292 cells. (A)** PCN is redox active, and induces the formation of ROS and RNS. **(B)** NCI-H292 cells were exposed to PCN at indicated concentrations in the presence or absence of GSH for 24 hr. For antioxidant treatment, NCI-H292 cells were prexposed to GSH for 60 min before PCN was added. The experiments were performed in triplicates for three times with similar results. The mean ± standard deviation (SD) from one typical experiment is shown. *p < 0.05 when comparing PCN-exposed cells against control cells exposed to 12.5 μl sterile water per milliliter of culture medium.

The human lung mucoepidermoid carcinoma cell line NCI-H292 was purchased from the American Type Culture Collection (ATCC) (Manassas, VA, USA). 16HBE cells [1] were a generous gift from Dr. D.C. Gruenert (University of California, San Francisco, CA). NCI-H292 and 16HBE cells were cultured in RPMI-1640 and MEM respectively, supplemented with 10% fetal bovine serum in 5% CO2. Epithelial cells that reached 70% confluency were serum-starved for 24 hr before exposure to indicated concentrations of PCN. As a control, cells were exposed to sterile H_2_O that corresponded to maximum volume of PCN used in each experiment. For example, 12.5 μl/ml sterile water was used per milliliter of culture medium in Figure 1B. Normal human bronchial epithelial (NHBE) cells were purchased from Lonza (Walkersville, MD, USA). Cells were passaged in 5% CO_2_ at 37°C using the bronchial epithelial growth medium (BEGM) supplemented with growth factors supplied in the SingleQuot® kit (Lonza). NHBE cells at passages 2 to 4, and 16HBE cells were trypsinized and seeded onto the Costar Transwells® inserts with 0.4 μm pore size (Corning) (Tewksbury, MA, USA) at a density of 1.5 × 10^5^ cells/cm^2^ in media comprised of 50% BEBM and 50% DMEM-F12 low glucose (Invitrogen, Grand Island, NY, USA) supplemented with the growth factors provided in the SingleQuot® kits and retinoic acid (50 nM). Once the cells reached confluency (approximately seven days after seeding), they were switched to an air-liquid interface for an additional 2 weeks to achieve mucociliary differentiation. PCN (12.5 μg/ml) or IL-13 (1 μg/ml) was added to the Transwell chambers for 24 hr. Sterile water was used as the control. NHBE cells were stained with mouse anti-MUC5AC monoclonal antibody (clone 45 M1, Santa Cruz Biotechnology, Santa Cruz, CA, USA, #sc-21701, dilution 1:500), and visualized with Alexa Fluor®488-conjugated secondary antibodies (green color) under a confocal microscope. Nuclei were stained with DAPI (blue color). Brightfield and fluorescence images of these cells can be found in the Additional file 1: Figure S1 and Additional file 2: Figure S2.

### ROS assays

ROS levels in PCN-exposed NCI-H292 cells were determined using the OxiSelect™ In Vitro ROS/RNS Assay Kit according to the manufacturer protocols (#STA-347, Cell Biolabs, San Diego, CA, USA). The assay uses the specific ROS/RNS probe dichlorodihydrofluorescin DiOxyQ (DCFH-DiOxyQ). The DCFH-DiOxyQ probe is first primed with a quench removal reagent, and subsequently stabilized in the highly reactive DCFH form. ROS and RNS species react with DCFH, which then rapidly oxidizes to the highly fluorescent 2’, 7’-dichlorodihydrofluorescein (DCF). Fluorescence intensity is proportional to the total ROS/RNS levels within the sample. The DCFH-DiOxyQ probe can react with hydrogen peroxide (H_2_O_2_), peroxyl radical (ROO·), nitric oxide (NO), and peroxynitrite anion (ONOO^-^), allowing for measurement of the total free radical population within a sample.

### Mucin analysis

NCI-H292 or 16HBE cells were stimulated with indicated concentrations of PCN for 24 hr. Cells were lysed by the M-PER Mammalian Protein Extraction Reagent (#78501, Thermo Scientific, Rockford, IL, USA) in the presence of the Halt Protease Inhibitor Cocktail (Thermo Scientific #87786). The protease inhibitors were incorporated because of prior reports of sensitivity of the anti-mucin antibodies to degradation with cell lysates [34,35]. The amounts of mucins in total cell lysates were determined by western blotting using specific antibodies against MUC5AC (Santa Cruz Biotechnology #sc-21701, dilution 1:1000) and MUC5B (Santa Cruz Biotechnology #sc-20119, dilution 1:1000) or by ELISA kits (USCN LIFE SCIENCE, Houston, TX, USA, MUC5AC #E90756Hu; MUC5B #E90684Hu). These ELISA kits have been previously used in mucin studies [36-38].

### Posttranslational modification of FOXA2

Nuclear proteins from PCN-stimulated or control NCI-H292 cells were purified using the NE-PER Nuclear and Cytoplasmic Extraction Reagents (Thermo Scientific #78835). FOXA2 was immunoprecipitated using anti-FOXA2 antibody (Santa Cruz Biotechnology #sc-101060) immobilized on Protein A/G Agarose (Thermo Scientific #89931). Posttranslational modifications of FOXA2 were analyzed by western blot using antibodies against nitrotyrosine (Santa Cruz Biotechnology #sc-101358, dilution 1:1000), acetylated lysine (Abcam Cambridge, MA USA, #ab80178, dilution 1:1000), methylated lysine (Abcam, #ab76118), and ubiquitin (Santa Cruz Biotechnology, #FL-76, dilution 1:1000).

### Neutralization of PCN by GSH

NCI-H292 cells were pretreated with GSH at indicated concentrations (0.2 to 10 mM) for 60 min before exposure to PCN or sterile H_2_O for 24 hr. Total or nuclear proteins were extracted for western blotting or ELISA. The levels of GSH in rodent lungs have been measured to be 2 mM [39]. GSH at concentrations as high as 10 mM has been used in cell cultures [40].

### Western blot analyses

Cytoplasmic or nuclear proteins were separated by 8% SDS-PAGE and transferred to PVDF membrane (Bio-Rad, Hercules, CA, USA, #162-0177). Membranes were probed with individual primary antibodies. The immune complexes were visualized by the HRP-conjugated anti-mouse or anti-rabbit secondary antibodies (1:5000 dilution) using the ECL Western Blotting Detection System (Amersham Biosciences, Piscataway, NJ, USA #RPN2132) on Kodak BioMax X-ray films (Kodak, Rochester, NY, USA, #Kodak XAR-51651454). The membranes were stripped and probed with anti-β-actin antibody (Santa Cruz Biotechnology #47778, dilution 1:2000) as loading control for MUC5AC and MUC5B or with anti-H3 antibody (Santa Cruz Biotechnology #sc-8655, dilution 1:2000) as control for FOXA2.

### EMSA

Nuclear extracts from PCN-treated and control NCI-H292 cells were immunoprecipitated with anti-FOXA2 antibody using the Crosslink Immunoprecipitation Kit (Thermo Scientific #26147). Immunoprecipitated FOXA2 free of antibody contamination (2.5 μg) was incubated with the biotin-labeled probes in incubation buffer (10 mM Hepes pH 7.9, 1 mM EDTA, 5 mM MgCl_2_, 10% glycerol, 0.5 m MDTT, 1 mg/ml BSA and 1 μg PolydI · dC) for 20 min at room temperature. The biotin-labeled probes used were: Forward 5′-CCTCCCCGAGAGCAAACACACGTGGCTGGA-3′, and Reverse: 5′-TCCAGCCACGTGTGTTTGCTCTCGGGGAGG-3′, corresponding to the FOXA2 binding site on *MUC5B* gene promoter. For competition assays, extracts were incubated with 20-fold excess of unlabeled probes or with anti-FOXA2 antibody. FOXA2-DNA complexes were separated on a 6% acrylamide gel, transferred to Hybond nitrocellulose membranes (Amersham Biosciences #RPN2020D), and developed using the LightShift Chemiluminescent EMSA Kit (Thermo Scientific #20148).

### Quantitative real-time polymerase chain reaction (qRT-PCR) analysis

NCI-H292 cells were cultured in 6-well plates and stimulated with 0, 3.125, or 12.5 μg/ml of PCN for 24 hr with or without pretreatments with 0.4, 1.0, or 2.5 mM concentrations of GSH for 60 min before exposure to PCN. Total RNA was extracted using the RNeasy Mini Kit (Qiagen # 74104) according to the manufacturer’s instructions. Equal amount of total RNA (3 μg) was reverse transcribed into cDNA using oligo(dT) primers and SuperScript III reverse transcriptase (Invitrogen). After the reverse transcription reaction, the first-stranded cDNA was then diluted and used in each subsequent PCR reaction. The qRT-PCR were performed on a 7900 HT real-time PCR system by using 10 μl of cDNA in the presence of Taqman primers predesigned by Applied Biosystems (Grand Island, New York) based on the sequence of the target genes, according to the manufacturer’s protocol. The relative expression of each gene was normalized to *GAPDH* to give a relative expression level. The primers information of *MUC5AC*, *MUC5B* and *GAPDH* genes are propriety information belonging to the Applied Biosystems. The Assay IDs for these primers are: *MUC5AC*: Hs01370716_m1, MUC5B: Hs 00861588_m1 and *GAPDH*: Hs 99999905_m1.

### Mouse lung infection and histopathological evaluation

C57BL6 mice (6-week old, Harlan Sprague Dawley, Indianapolis, IN, USA) were housed in positively-ventilated microisolator cages with automatic recirculating water, located in a room with laminar, high efficiency particle accumulation–filtered air. The animals received autoclaved food, water, and bedding. Mice (8/group) were anesthetized with isoflurane, and intranasally-infected with 1 × 10^6^ wild-type *PA* strain PAO1 or isogenic ∆*phzS* bacteria [30] on Day 1, 3, 5 and 7. Mouse lungs were collected on Day 8 for histopathological analyses as we previously published [31,33]. Briefly, a cannula was inserted in the trachea, and the lung was instilled with 10% neutral-buffered formalin at a constant pressure (25 cm H_2_O). The trachea was ligated, and the inflated lung was immersed in 10% neutral buffered formalin for 24 hr before being embedded in paraffin wax. Paraffin-embedded sections (4–8 μm thickness) were stained with periodic acid-Schiff’s reagent (PAS). The pathological inflammation, GCHM, and mucin expression from the midsagittal section of the lungs were evaluated under a light microscope. For immunohistochemistry (IHC) analyses, mouse lung sections were stained with primary antibodies and visualized using the VECTASTAIN Elite ABC Kit (Rabbit IgG, Vector Laboratories, Burlingame, CA, USA #PK-6101) (for MUC5B) or the Mouse on Mouse (M.O.M.) Elite Peroxidase Kit (#PK-2200) (for MUC5AC), according to protocols supplied by the manufacturers. The pathological inflammation and GCHM in a midsagittal section from the lung were evaluated under light microscope. A blinded pathologist in the Department of Pathobiology, University of Illinois at Urbana-Champaign (UIUC) independently examined the tissue sections. Animal studies were carried out in strict accordance to the protocol approved by the Institutional Animal Care and Use Committee (Protocol #11158) at the UIUC.

### Statistical analysis

Parametric data were analyzed for statistical significance by Student’s *t*-tests, with differences between means considered significant when *p*-value < ·0.05.

## Results

### PCN causes ROS/RNS stress in NCI-H292 cells

PCN damages airway epithelial cells by causing oxidative stress through the release of ROS (Figure 1A). Because H_2_O_2_ and O_2_ ^-^ are capable of interacting with NO within airways to produce the highly toxic peroxynitrite, we evaluated the production of total ROS/RNS in NCI-H292 cells following 24 hr exposure to different concentrations of PCN. PCN caused a dose dependent increase of ROS/RNS (Figure 1B). For example, at 3.125 μg/ml, PCN only caused a slight increase in ROS/RNS. However, at clinically relevant concentrations of 6.25 and 12.5 μg/ml, PCN significantly increased the production of ROS/RNS by 47 and 50%, respectively. We also examined whether GSH could attenuate the ROS/RNS production. GSH is a ubiquitous, essential tripeptide (L-g-glutamyl-L-cysteinyl-glycine) antioxidant containing a sulfhydryl group that enables it to protect against oxidant-induced lung injury and inflammation [41]. Importantly, pretreatment of NCI-H292 cells for 60 min with the antioxidant GSH (≥ 1 mM) before the exposure to PCN limited the ROS/RNS production to basal levels (Figure 1B). These results suggest that ROS/RNS induced by PCN may impair the function of various host proteins in the airway epithelial cells. However, GSH efficiently neutralizes PCN toxicity.

### PCN induces posttranslational modifications and degradation of FOXA2

**Figure 2:**
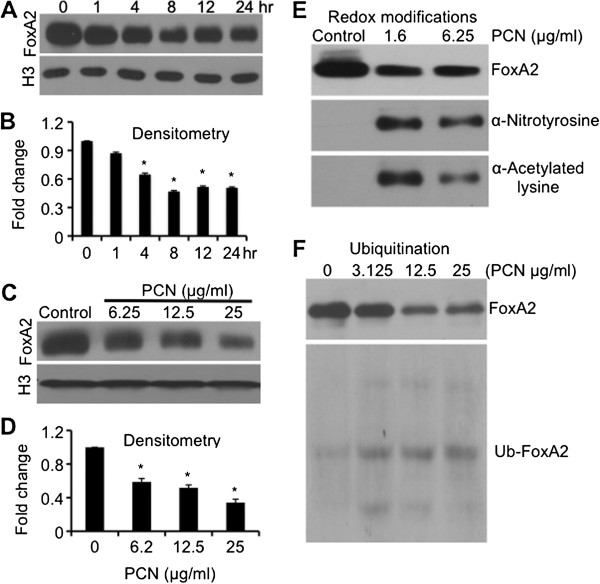
**PCN induces posttranslational modifications and degradation of FOXA2.** FOXA2 proteins were immunoprecipitated from nuclear lysate of NCI-H292 cells previously exposed to PCN for indicated times and concentrations, using anti-FOXA2 monoclonal antibody immobilized on Protein A/G Agarose, and analyzed by western blots using specific antibodies. The experiments were repeated three times with similar results. One typical western blot is shown. Densitometry data represent the mean ± SD from all three experiments. **(A-D)** Time and concentration-dependent inhibition of FOXA2 expression by PCN. **(E-F)** Decreasing levels of FOXA2 is accompanied by increasing levels of nitrosylation and acetylation **(E)** and ubiquitination **(F)**.

FOXA2 is required for maintenance of normal differentiation of the airway epithelium [32]. Inhibition of FOXA2 by pro-GCHM Stat6 and EGFR signaling pathways appears to be an important early step in the initiation of GCHM and mucus hypersecretion [32,42,43]. We have previously shown that PCN causes GCHM and mucus hypersecretion by inhibiting the expression of FOXA2, primarily through the activation of Stat6 and EGFR [31,33]. However, because ROS/RNS directly damage proteins, we examined whether PCN-generated ROS/RNS induced posttranslational modifications of FOXA2, resulting in its degradation in the NCI-H292 cells. PCN caused a time and concentration-dependent inhibition of FOXA2 expression (Figure 2A-D). Furthermore, western blotting analyses suggest that FOXA2 in the nuclei of NCI-H292 cells exposed to PCN undergo nitrosylation and acetylation (Figure 2E) and ubiquitination (Figure 2F). Ubiquitination of FOXA2 suggests that it was destined for degradation. This is consistent with the finding that increasing levels of nitrosylation and ubiquitination is accompanied by decreasing levels of FOXA2 following treatment with PCN (Figure 2E, F). Interestingly, we were not able to detect a significant increase in the level of FOXA2 oxidation, methylation or thiolation (data not shown). Collectively, these results suggest that PCN-generated ROS/RNS posttranslationally modify FOXA2, leading to its ubiquitination and degradation.

### FOXA2 posttranslationally modified by PCN has reduced ability to bind to the promoter of MUC5B gene

**Figure 3:**
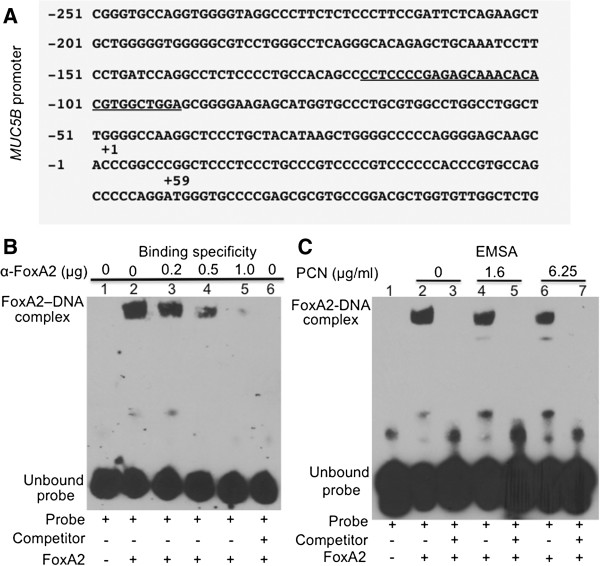
**Posttranslational modification of FOXA2 by PCN reduces its binding to the promoter of *MUC5B *gene. (A)** FOXA2 binding site (underlined) on the *MUC5B* promoter. **(B)** Pre-incubation with anti-FOXA2 antibody blocks the binding of FOXA2 to MUC5B promoter, suggesting that the MUC5B-promoter binding is FOXA2-specific. Biotin-labeled probes were incubated alone (Lane 1), or with equal amounts of FOXA2 immunoprecipitated from NCI-H292 cells in presence of indicated amount of anti-FOXA2 antibody (Lanes 2–5) or a 20-fold excess of unlabeled probe (Lane 6). **(C)** FOXA2 immunoprecipitated from PCN-exposed NCI-H292 cells has reduced ability to bind to the promoter of *MUC5B* gene. Biotin-labeled probes were incubated alone (Lane 1) or with equal amounts of FOXA2 immunoprecipitated from PCN exposed NCI-H292 cells in the absence (Lanes 2, 4 and 6) or in the presence of a 20-fold excess of unlabeled probe (Lanes 3, 5 and 7).

Our experimental evidence indicates that FOXA2 is posttranslationally modified by ROS/RNS produced by redox activities of PCN. Modified FOXA2 may lose its transcriptional repressor activity, leading to GCHM and derepression of mucin biosynthesis genes. MUC5B, MUC5AC and MUC2 are major secreted mucins in the airway mucus [44]. MUC5AC and MUC5B are abnormally augmented in airway disease states, such as chronic bronchitis, COPD, asthma and CF [44]. However, studies have shown that MUC5B is the predominant mucin in the CF and COPD airways [45,46]. We used EMSA to examine the ability of ROS/RNS-modified FOXA2 to bind the promoter of the *MUC5B* gene (Figure 3A) in the NCI-H292 cells. Because PCN inhibits the expression and induces degradation of FOXA2, EMSA assays were performed using equal amounts of FOXA2 protein immunoprecipitated from control and PCN-exposed NCI-H292 cells. Immunoprecipitated FOXA2 proteins free of antibody were allowed to complex with biotin-labeled DNA oligos alone or in the presence of excess non-biotin-labeled competitor. The specificity of the FOXA2 binding to the promoter of *MUC5B* was confirmed by a competition assay with unlabeled probe and with antibodies against FOXA2 (Figure 3B). FOXA2-DNA complex formation was inhibited by 20-fold excess of competitor probe (Figure 3B, Lane 6; Figure 3C, Lanes 3, 5, 7), and by increasing the amounts of anti-FOXA2 antibody (Figure 3B, Lanes 2, 3, 4, 5). As shown in Figure 3C, FOXA2–DNA interaction was observed when the FOXA2 extracts were incubated with biotin-labeled probes (Lanes 2, 4 and 6). However, decreasing amounts of FOXA2-DNA complexes were detected when FOXA2 was immunoprecipitated from NCI-H292 cells exposed to 1.6 μg/ml PCN and 6.25 μg/ml PCN treatment (Lanes 4 and 6) compared to the control (Lane 2). Collectively, these results suggest that PCN-generated ROS/RNS posttranslationally modify FOXA2, impairing its ability to effectively bind to the promoter of the *MUC5B* gene.

### PCN induces MUC5AC and MUC5B expression

**Figure 4:**
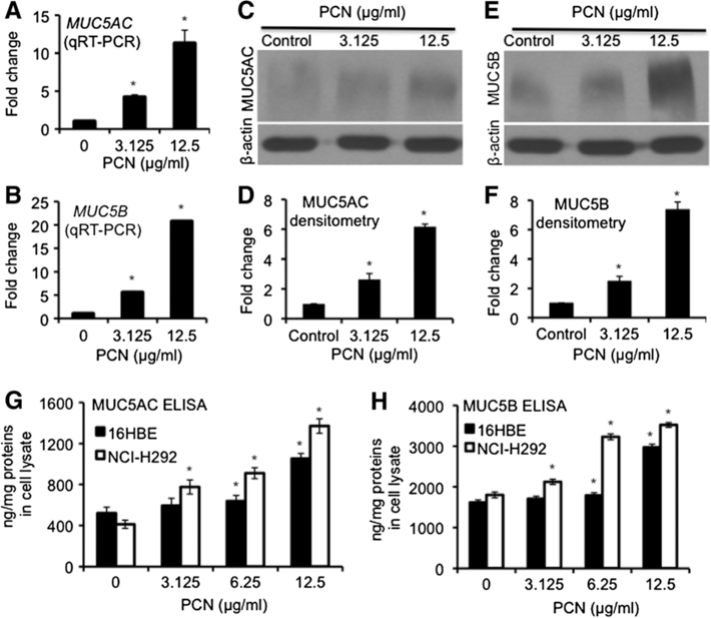
**PCN induces the expression of mucins in NCI-H292 and 16HBE airways epithelial cells. (A**-**D)** NCI-H292 cells were exposed to PCN at indicated concentrations for 24 hr. **(A**-**B)** qRT-PCR of *MUC5AC* and *MUC5B* genes in the presence of PCN. **(C**-**F)** Western blotting and densitometry analyses of the expression of both MUC5AC **(C**-**D)** and MUC5B **(E**-**F)**. The same membranes were stripped and probed with antibody against β-actin for loading controls. **(G**-**H)** Expression of MUC5AC **(G)** and MUC5B **(H)** mucins in NCI-H292 and 16HBE cells after 24 hr of exposure to clinically relevant concentrations of PCN, and quantified by ELISA. The qRT-PCR and ELISA experiments were performed in triplicates in three independent experiments. The western blotting experiments were repeated three times with similar results. Data for qRT-PCR, ELISA and densitometry of western blots represent the mean ± SD from all three experiments. *p < 0.05 when PCN-exposed cells were compared with the control cells exposed to same volume of sterile water.

**Figure 5:**
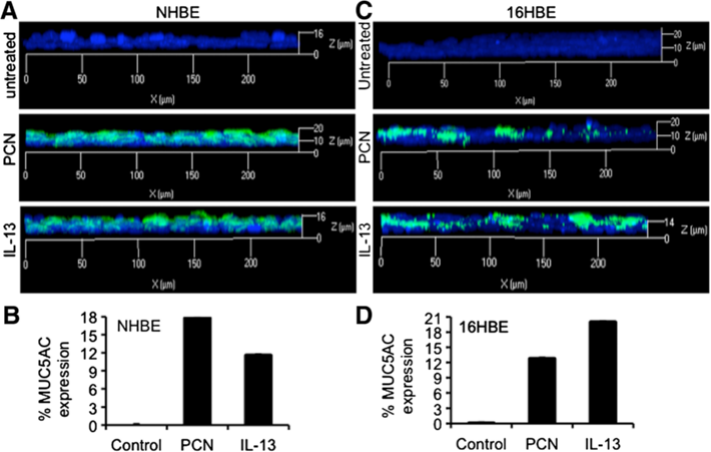
**PCN induces the expression of mucins in polarized NHBE and 16HBE airways epithelial cells. (A-D)** Induction of MUC5AC mucin expression in polarized NHBE **(A)** and 16HBE **(C)** cells cultured at the air-liquid interface by PCN (12.5 μg/ml). Epithelial cells exposed to IL-13 (1 μg/ml) or sterile water (12.5 μl/ml) were used as positive or negative control for MUC5AC induction, respectively. MUC5AC induction by PCN **(B)** and IL-13 **(D)** was quantified by the ratio of MUC5AC fluorescence signal (green) divided by the total fluorescence signal of DAPI stained nuclei (blue) using the AxioVision Rel. 4.8 software.

As shown above, FOXA2 protein extracted from NCI-H292 cells previously exposed to PCN has posttranslational modifications and impaired binding to the promoter of the MUC5B gene. These observations suggest that posttranslationally modified FOXA2 may have reduced ability to repress the expression of mucin genes. We investigated the derepression of the transcription of *MUC5B* and *MUC5AC* genes, as well as the biosynthesis and secretion these mucins in lung epithelial cells after treatment with PCN, by qRT-PCR, western blotting, ELISA and immunofluorescence. Previously, we have shown that PCN significantly induced MUC5B expression in human primary bronchial epithelial (NHBE) cells and in 16HBE cells cultured at the air-liquid interface [33]. In the presence of 12.5 μg/ml of PCN, qRT-PCR analyses revealed that the expression of *MUC5AC* and *MUC5B* genes were increased significantly by 11 and 21-fold, respectively (Figure 4A-B). Densitometry analyses of western blots indicate that the expression of MUC5AC and MUC5B proteins increased by 4 and 5-fold, respectively (Figure 4C-F). These results were confirmed by ELISA analyses, which showed dose-dependent induction of both MUC5AC and MUC5B mucins by PCN in both NCI-H292 and 16HBE cells (Figure 4G-H). It is also apparent that MUC5B was expressed in higher concentrations both in the presence and absence of PCN, but the level of induction by PCN was similar between the two mucins (compared Figure 4G to 4H). Immunofluorescence staining indicated that, similar to MUC5B [33], PCN induced the expression of MUC5AC (green fluorescence) in NHBE (Figure 5A-B) and 16HBE (Figure 5C-D) cells cultured at the air-liquid interface to similar extent as the positive control IL-13.

### PCN-deficient PA mutant is attenuated in its ability to induce the goblet cell hyperplasia and metaplasia in mouse airways

**Figure 6:**
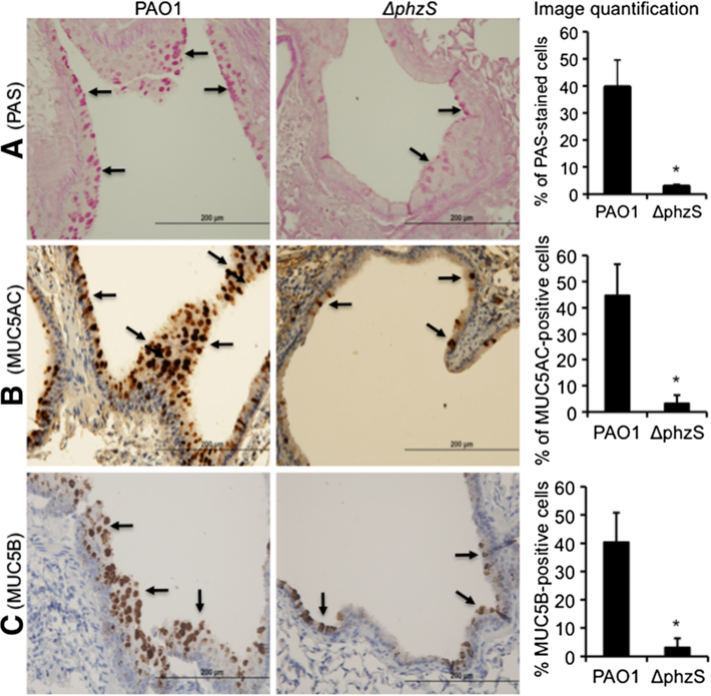
**PCN-deficient mutant has reduced ability to induce the goblet cell hyperplasia in mouse airways.** C57BL6 mice (n = 8) were repeatedly challenged with 1 x 10^6^ of wild-type *PA* PAO1 or the isogenic ∆*phzS* mutant on Day 1, 3, 5 and 7. Mouse lungs were harvested, fixed and paraffin-embedded for PAS staining and for IHC. **(A)** PAS staining and quantification of goblet cells after infection by PAO1 versus ∆*phzS*. **(B)** IHC and quantification of MUC5AC induction by PAO1 versus ∆*phzS*. **(C)** IHC and quantification of MUC5B induction by PAO1 versus ∆*phzS* mutant. PAS, MUC5AC and MUC5B-positive cells were quantified using the AxioVision Rel. 4.8 software. Five representative microscopic fields at 10x magnification from each mouse were quantified. Data represent the mean ± SD from all eight mice.

We have previously shown that chronic exposure to PCN induces GCHM and mucus hypersecretion [31,33]. However, no studies thus far have comparatively examined the induction of GCHM and mucus secretion by wild-type *PA* versus PCN-deficient mutant. C57BL6 mice were repeatedly challenged with 1 × 10^6^ of wild-type *PA* PAO1 or the isogenic PCN-deficient ∆*phzS* mutant on Day 1, 3, 5 and 7. All eight mice challenged with the wild-type PAO1 developed robust GCHM and mucus hypersecretion as indicated by PAS-stained mucins (Figure 6A-C, arrows). In contrast, only one out of eight mice infected with the ∆*phzS* mutant showed low levels of isolated mucin-expressing goblet cells (Figure 6A). IHC analyses indicate that the expression of MUC5AC (Figure 6B) and MUC5B (Figure 6C) mucin were significantly higher in PAO1-infected airways when compared to the ∆*phzS* infected airways. These results concur with the results from *in vitro* studies in NCI-H292 and 16HBE cells, and *ex vivo* studies using NHBE cells, which indicate that PCN is a strong inducer of GCHM and mucus hypersecretion in airways.

### GSH alleviates the RNS-mediated FOXA2 modification and degradation

**Figure 7:**
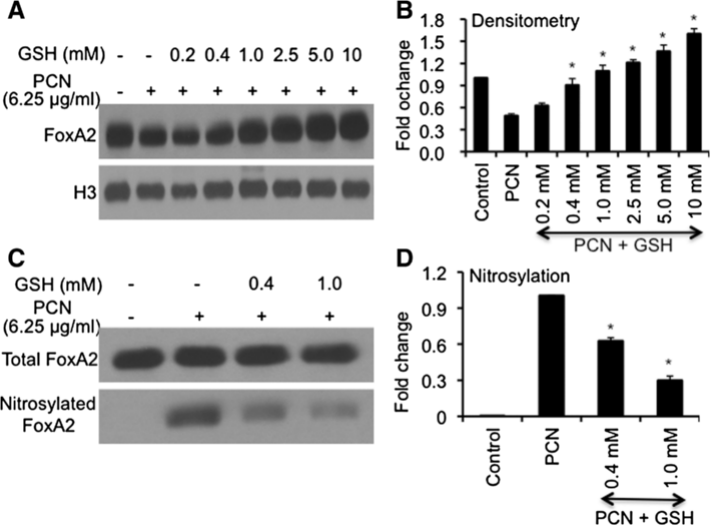
**GSH attenuates the repression and nitrosylation of FOXA2 by PCN. (A-B)** GSH treatment overcomes PCN-mediated inhibition of FOXA2 expression. NCI-H292 cells were pretreated with various concentrations of GSH for 60 min before exposure to PCN (6.25 μg/ml). Nuclear proteins were harvested for Western blot using anti-FOXA2 **(A)** and quantified with densitometry **(B)**. The same membrane was stripped and probed with antibody nuclear protein Histone H3 for loading control*.***(C-D)** GSH reduces nitrosylation of FOXA2 by PCN. NCI-H292 cells were pretreated with PBS (negative control) or with GSH for 60 min before exposing to PCN. FOXA2 was immunoprecipitated from nuclear extracts, and equal amounts were loaded for western blot analyses using anti-FOXA2 and anti-nitrotyrosine antibodies, respectively **(C)**. FOXA2 nitrosylation was quantified by densitometry **(D)**. The experiments were independently performed three times with similar results. Western blots from one typical experiment are shown. Densitometry data represent the mean ± SD from all three experiments. *p < 0.05 when comparing GSH-pretreated cells against cells exposed only to PCN.

Next, we examined whether the antioxidant GSH could attenuate the toxicity of PCN-generated ROS/RNS. We postulated that GSH could relieve the suppression and reduce nitrosylation of FOXA2 in the NCI-H292 cells. As shown in Figure 7A, PCN (6.25 μg/ml) reduced the expression of FOXA2 by 43%. However, GSH restored the expression of FOXA2 in a concentration dependent manner. At concentrations of ≥ 1 mM, GSH increased the expression of FOXA2 in PCN-exposed NCI-H292 cells above untreated control (Figure 7A-B). In addition, GSH also reduced the posttranslational modification of FOXA2. The levels of nitrosylated FOXA2 decreased significantly by 40% and 76% in the presence of 0.4 mM and 1 mM GSH, respectively (Figure 7C-D). Collectively, these results suggest that higher levels of antioxidants in airway epithelial cells can reduce posttranslational modification and inactivation of FOXA2-mediated by PCN-generated ROS/RNS. Furthermore, antioxidant treatment may enhance the expression of FOXA2.

### GSH treatment relieves the suppression of FOXA2 and represses mucin production induced by PCN

**Figure 8:**
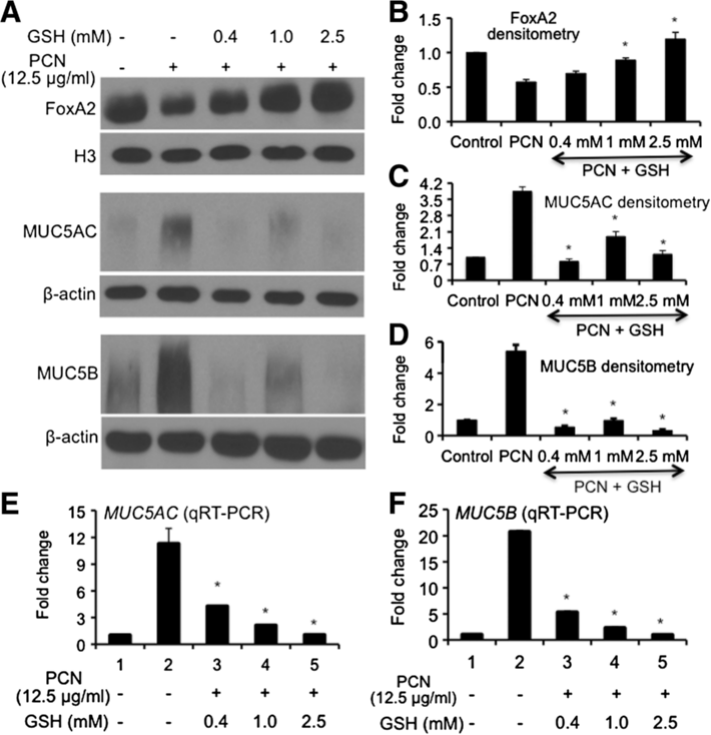
**Restoration of FOXA2 expression by GSH attenuates PCN-mediated overexpression of MUC5AC and MUC5B mucins. (A-D)** GSH treatment restores the expression of FOXA2 and attenuates the expression of MUC5AC and MUC5B mucins. NCI-H292 cells with or without GSH pretreatment were exposed to PCN (12.5 μg/ml) or sterile water (control) for 24 hr. Nuclear proteins were harvested for Western blots using anti-FOXA2 whereas total protein was probed with anti-MUC5AC or MUC5B antibodies **(A)** and quantified with densitometry **(B-D)**. The same membranes were stripped and probed with antibody nuclear protein Histone H3 for loading control for FOXA2 and β-actin as loading control for MUC5AC and MUC5B. The experiments were independently repeated three times. Densitometry data represent the mean ± SD from three experiments. *p < 0.05 when comparing GSH-pretreated cells against cells exposed only to PCN. **(E-F)** GSH represses PCN-mediated induction of *MUC5AC* and *MUC5B* gene expression. The expression of mucin genes was analyzed by qRT-PCR. The experiments were performed in triplicates in three independent experiments. The data represent the mean ± SD from three experiments.

Because the epithelial cells treated with GSH overcome the repression of FOXA2 expression and reduce posttranslational modification by PCN, we postulated that restored FOXA2 in turn, could inhibit the expression of MUC5AC and MUC5B mucins in the NCI-H292 cells. Western blot analyses showed that in the absence of GSH, PCN (12.5 μg/ml) reduced the expression of FOXA2 by 50%, with corresponding 5-fold increase in MUC5AC and MUC5B expression (Figure 8A-D). Addition of GSH significantly increased the expression of FOXA2, with corresponding decrease in the expression of MUC5AC and MUC5B mucins (Figure 8A, C-D). Restored expression of FoxA2 is also associated with repression of *MUC5AC* and *MUC5B* transcription (Figure 8E-F). Collectively, these results suggest that GSH effectively neutralizes PCN toxicity, restoring FOXA2 function, which in turn, may repress the transcription of *MUC5AC* and *MUC5B* genes as well as the expression of both mucins in airway epithelial cells.

## Discussion

PCN is a redox-active virulence factor of *PA.* We have previously shown that PCN inhibits the expression of FOXA2 through the activation of pro-GCHM signaling pathway Stat6 and EGFR [33]. In this study, we demonstrate PCN-generated ROS/RNS causes posttranslational modifications–nitrosylation, acetylation, and ubiquitination–of FOXA2, resulting in degradation of the transcriptional repressor of GCHM. Furthermore, FOXA2 modified by PCN-generated ROS/RNS has reduced binding capacity to the promoter of the *MUC5B* gene. The loss of FOXA2 expression is positively correlated to derepression of *MUC5AC* and *MUC5B* transcripts, as well as the overexpression of both mucins. Importantly, the antioxidant GSH neutralizes PCN-mediated toxicity and reduces the nitrosylation and suppression of FOXA2 by PCN-generated ROS/RNS. Restoration of FOXA2 expression is positively correlated to the repression of both *MUC5AC* and *MUC5B* genes and mucins. Collectively, these results suggest that posttranslational modification and inactivation of FOXA2 induced by PCN-generated ROS/RNS may also contribute to GCHM and mucus hypersecretion.

There has been a continual debate as to the importance of PCN to the pathogenesis of diseases in human airways, especially in CF. This is primarily because of conflicting levels of PCN that were recovered within a limited number of sputum samples from CF and non-CF bronchiectatic patients [12]. In some of the samples, PCN levels as high as 27 and 16.5 μg/ml were recovered, the latter of which, was from a CF patient. However, a few other samples showed either low or non-detectable levels of PCN. Thus, a new study involving a larger cohort of patients is needed. In addition, one recent study has shown that some PCN-deficient CF isolates of *PA* are associated with BPI-ANCA and progressive lung disease, suggesting that toxin-mediated alterations are not important for infection in this subpopulation of CF patients [47]. However, it is important to note that most of the CF clinical isolates of *PA* secrete more PCN *in vitro*[31]. Furthermore, PCN is overproduced by laboratory strains grown in minimal medium supplemented with CF sputum rather than glucose [13]. In addition, overproduction of PCN has been reported among the hypervirulent Liverpool Epidemic CF strains of *PA*[48]. More importantly, PCN hypersecretion was correlated with episodes of pulmonary exacerbations in a set of CF patients [49]. We have previously shown that PCN is important for acute and chronic infection of mouse airways [30,31]. Additional evidence of the importance of PCN during *PA* infection include both *in vitro* and *in vivo* models of infection or intoxication, and the induction of GCHM and mucus hypersecretion [31,33]. The results from our current study provide additional supporting evidence of the involvement of PCN in both induction and exacerbation of GCHM and mucus hypersecretion in CF and non-CF bronchiectatic and COPD airways chronically infected by *PA* strains producing PCN.

Apart from PCN, other virulence factors of *PA*, including LPS are known to induce oxidative stress [50]. However, as we discussed earlier, the O-antigen of *PA* LPS is frequently mutated [7]. In addition, ~40% of *PA* isolates are non-flagellated, especially in mucoid isolates that reside in the chronic CF airways [6]. Comparative studies between the wild-type *PA* strain PAO1 and its isogenic ∆*phzS* mutant indicate that inability to synthesize PCN hampers the ability of *PA* to induce GCHM and mucus hypersecretion. Thus, PCN appears to be an important inducer of ROS/RNS, which contributes to mucus hypersecretion in diseased airways chronically infected by *PA*. This argument is supported by studies showing that ROS/RNS play a prominent role in the pathogenesis of acute lung injury, ARDS, interstitial lung disease, CF, COPD and asthma [51-54], including the recent clinical data suggesting that oxidative damage of pulmonary proteins during chronic infection may contribute to the decline of lung function in CF patients [52]. These clinical findings are consistent with the FOXA2 inactivation by PCN-generated ROS/RNS, which may contribute and exacerbate GCHM and mucus hypersecretion in diseased airways colonized by *PA*.

Previously, we have demonstrated that PCN can inhibit the expression of FOXA2 through the activation of IL-4/IL-13/Stat6 and EGFR signaling pathways [33]. Especially relevant is the finding that EGFR, a major pro-GCHM pathway, is inducible by ROS [55], including those generated by PCN [33,56]. Thus, PCN-mediated GCHM and mucus hypersecretion in diseased airways chronically colonized by *PA* is likely a cumulative effect of ROS/RNS-mediated posttranslational modification of FOXA2 and the activation of IL-4/IL-13-STAT6 and EGFR signaling pathways that inhibit the expression of FOXA2. However, excessive levels of PCN-generated ROS/RNS may potentially impair the function of components of Stat6 and EGFR pathways. Thus, it is likely that PCN-mediated repression of FOXA2 is a bigger contributor of GCHM and mucus hypersecretion than the partial loss of function for both Stat6 and EGFR signaling components. Future studies will address these matters in more detail.

Thiol compounds, including GSH, are known to play important roles in pulmonary diseases [41]. One study in rats has shown that the concentration of lung GSH is approximately 2 mM [39]. GSH at concentrations as high as 10 mM has been used in cell culture experiments [40]. Importantly, inhaled GSH therapy has been shown to improve the lung function of CF patients, as well as liquefy the mucus [57-59]. Our results provide a possible mechanistic basis for the beneficial treatment with thiols. For example, GSH at physiologically relevant concentrations of 0.4 – 2.5 mM not only reduces the production of PCN-generated ROS/RNS, it also attenuates the posttranslational modifications and inactivation of FOXA2, and, in the process, suppresses the production of excessive mucins. Collectively, our experimental results suggest that providing physiologically relevant concentrations of GSH can counter the induction of GCHM by clinically relevant concentrations of PCN (≤ 25 μg/ml). Future studies will examine the efficacy of GSH against GCHM and mucus hypersecretion mediated by PCN in mouse airways, as well as during infection by wild-type versus PCN-deficient laboratory and clinical strains of *PA*.

One surprising finding from our studies is the robust mucin secretion induced by PCN in the polarized NHBE cells. At a clinically relevant concentration of 12.5 μg/ml, PCN induces higher levels of MUC5AC expression than IL-13 (1 μg/ml) after 24 hr of exposure in NHBE cells. Similarly, PCN also induces substantial expression of MUC5B mucin in the polarized NHBE cells [33]. Interestingly, induction of mucin secretion with short exposure to IL-13 has not been reported. One potential discrepancy between the current study and other reports lies in the IL-13 concentration used. We exposed NHBE cells to 1 μg/ml of recombinant human IL-13 and probed for mucin expression at 24 hr [31,33] whereas other studies used 10 ng/ml over 7–14 days to induce differentiation of mucous cells and increases in MUC5AC protein [60-62]. However, there have been other short duration studies that used higher concentrations of IL-13 to induce GCHM and mucin expression. For example, instillation of mouse recombinant IL-13 (250 ng per/mouse, twice over 48 hr; or 500 ng/mouse, once in 24 hr) has been shown to induce GCHM and mucin overexpression within mouse airways [63,64]. Thus, our current study resembles the studies that used higher amounts of IL-13 in short duration of exposure.

We acknowledge limitations to the interpretation of the results derived from the current study. Even though we have combined various techniques, including ELISA, immunohistochemistry, immunofluorescence, western blot and qRT-PCR to examine the impact of PCN on the expression of FoxA2 and mucin genes, a large portion of the data is based on *in vitro* analyses in immortalized cell lines. In addition, densitometry analysis of western blot is semi-quantitative and has limited sensitivity. Another limitation is on the mechanistic aspects of this study. We have shown that PCN-mediated posttranslational modifications of FOXA2 is positively associated with GCHM and upregulation of MUC5AC and MUC5B genes and mucins. Directly demonstrating that these posttranslational modifications of FOXA2 inactivate its function and cause GCHM and mucin hypersecretion remain unproven, and difficult. Additional experiments to unravel the mechanisms by which PCN-generated ROS/RNS posttranslationally modify and inactivate FOXA2 may include the use of mass spectrometry to map the amino acid residues modifies by ROS/RNS. This will be followed by site-directed mutagenesis and constructing various versions of mutated FOXA2 recombinants, and studying the resistance or susceptibility of these genetically altered FOXA2 recombinants to ROS/RNS-mediated posttranslational modifications and mucin gene regulation in both airway epithelial cells and in mouse lungs.

In summary, the present study shows that PCN down-regulates the expression of FOXA2 through posttranslational modifications mediated by ROS/RNS. Modified FOXA2 is degraded, as well as having reduced ability to bind the promoter of *MUC5B* gene. The degradation and functional impairment of FOXA2 is positively correlated to elevation of GCHM and mucin biosynthesis. Thus, inhibition of PCN biosynthesis and neutralization of its toxicity, and maintenance of FOXA2 function in diseased airways chronically infected by *PA* may be therapeutically useful to improve the lung functions of these patients.

## Abbreviations

PA: Pseudomonas aeruginosa; PCN: Pyocyanin (PCN); FOXA2: Forkhead box A2; GCHM: Goblet cell hyperplasia and metaplasia; ROS: Reactive oxygen species; RNS: Reactive nitrogen species; EMSA: Electrophoretic mobility shift assay; GSH: Glutathione; NHBE: Human primary bronchial epithelial.

## Competing interests

None of the authors has a financial relationship with a commercial entity that has an interest in the subject of this manuscript.

## Authors’ contributions

YH and ZZK contributed equally to this work. YH, ZZK and GWL designed the experiments. YH, ZZK, YX and GWL performed the experiments and analyzed the data. YH, ZZK, BEW and GWL wrote the manuscript. All authors read and approve the final manuscript.

## Supplementary Material

Additional file 1: Figure S1 Brightfield and fluorescence images of polarized NHBE cells cultured at the air-liquid interface after 24 hr of treatment with sterile H_2_O (control), PCN, IL-13. Click here for file

Additional file 2: Figure S2 Brightfield and fluorescence images of polarized 16HBE cells cultured at the air-liquid interface after 24 hr of treatment with sterile H_2_O (control), PCN, IL-13. Click here for file
