# Supplementary figures and images for: Pyocyanin-induced mucin production is associated with redox modification of FOXA2
Source: Respir Res. 2013 Aug 5;14(1):82. doi: 10.1186/1465-9921-14-82 (PMC3765780; doi:10.1186/1465-9921-14-82)

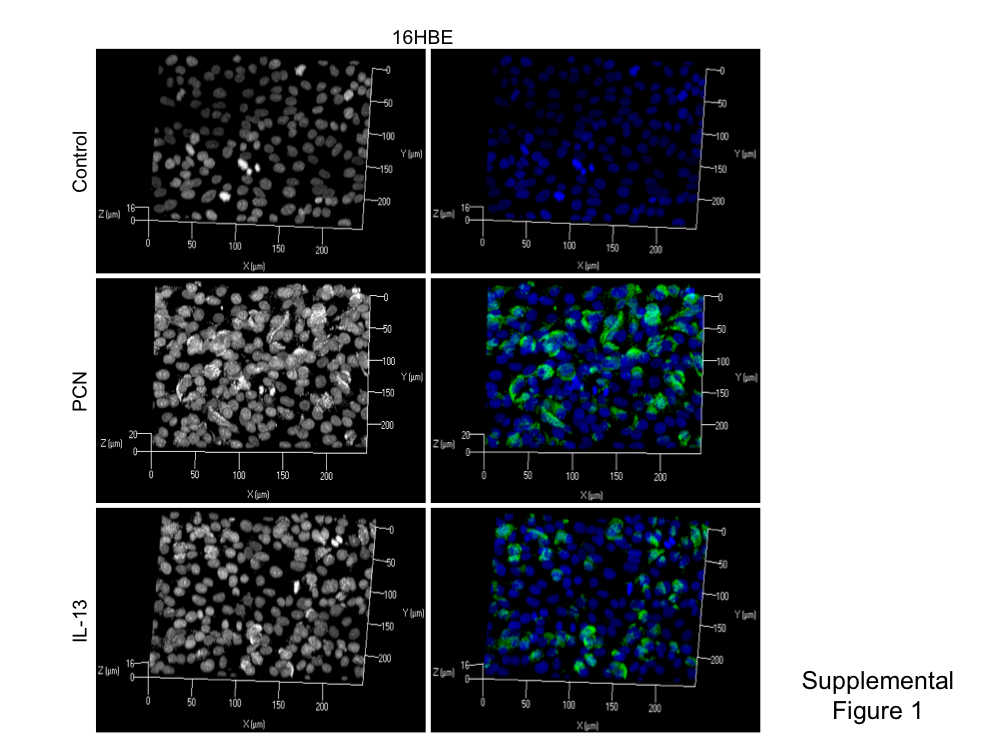

Supplement: Additional file 1: Figure S1 — Brightfield and fluorescence images of polarized NHBE cells cultured at the air-liquid interface after 24 hr of treatment with sterile H2O (control), PCN, IL-13. [file 1465-9921-14-82-S1.tiff]

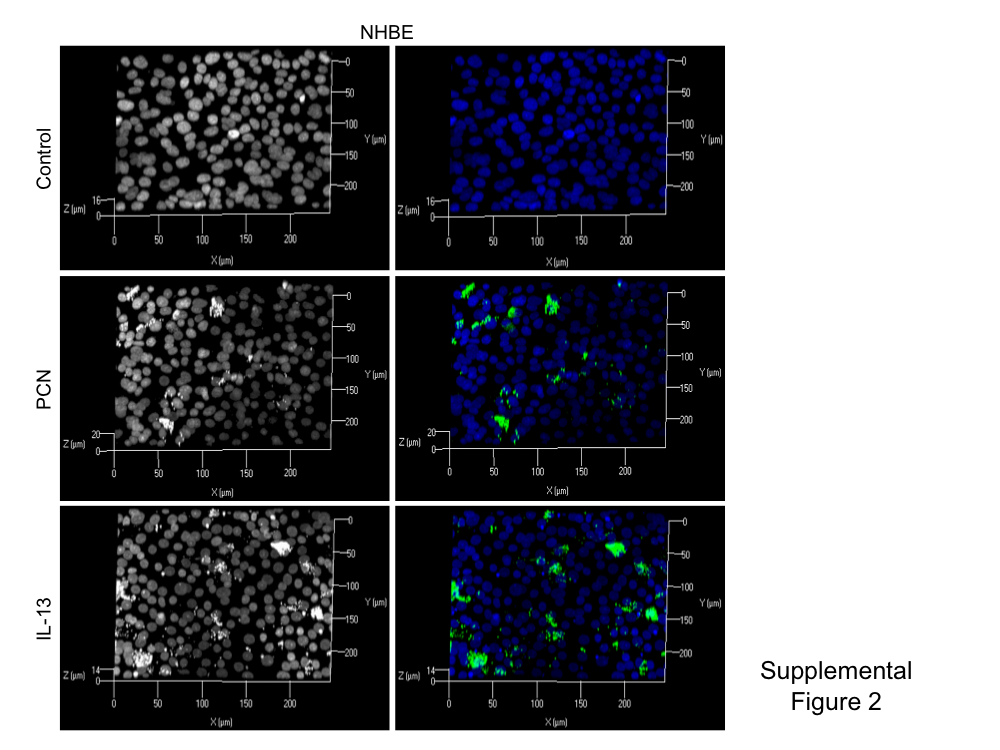

Supplement: Additional file 2: Figure S2 — Brightfield and fluorescence images of polarized 16HBE cells cultured at the air-liquid interface after 24 hr of treatment with sterile H2O (control), PCN, IL-13. [file 1465-9921-14-82-S2.tiff]
